# Supplementary material for: Parenteral Nutrition in the Pediatric Oncologic Population: Are There Any Sex Differences?
Source: Nutrients. 2023 Aug 31;15(17):3822. doi: 10.3390/nu15173822 (PMC10490019; doi:10.3390/nu15173822)
Supplement: Supplementary file 1 [file nutrients-15-03822-s001.zip › nutrients-2534248-supplementary.pdf]

SUPPLEMENTS

Table S1: Descriptive population analysis and the composition of TPN bags in the HSCT and non-HSCT groups.

	HSCT group			non-HSCT group		
**Characteristic**	**Males** (n = 126)[1]	**Females** (n = 79)[1]	**p-value**[2]	**Males** (n = 38)[1]	**Females** (n = 30)[1]	**p-value**[2]
**Age** (years)	9.0 (4.0, 15.0)	12.0 (9.0, 14.0)	0.06	10.0 (4.0, 14.8)	11.5 (5.2, 13.0)	>0.9
**Age group**			0.4			>0.9
>12 years	45 (36%)	33 (42%)		14 (37%)	11 (37%)	
0-12 years	81 (64%)	46 (58%)		24 (63%)	19 (63%)	
**TPN duration** (days)	12 (7, 20)	16 (9, 24)	0.078	7 (4, 11)	6 (4, 9)	0.2
**HSCT type**			0.3			
1- Autologous	42 (34%)	22 (28%)	0.4			
2- Allogenic	28 (22%)	12 (15%)	0.2			
3- MUD	47 (38%)	36 (46%)	0.2			
4- Haploidentical	8 (6.4%)	9 (11%)	0.2			
Unknown	1	0				
**Weight (kg)**	33 (19, 56)	41 (25, 51)	0.7	30 (18, 44)	42 (20, 52)	0.8
Unknown	13	9		6	5	
**Total number of TPN bags**	13 (7, 20)	15 (10, 23)	0.11	7 (4, 12)	6 (4, 9)	0.2
**Volume/day** (mL)	1,833 (1,150, 2,400)	1,927 (1,273, 2,435)	0.8	1,750 (1,275, 2,200)	2,000 (1,425, 2,500)	0.3
**Magnesium/day** (mg)	209 (102, 352)	214 (151, 358)	0.5	172 (99, 241)	186 (133, 219)	0.6
**Phosphorus/day** (mg)	283 (145, 478)	389 (166, 558)	0.12	392 (293, 538)	400 (313, 600)	0.4
**Calcium/day** (mg)	276 (132, 479)	181 (81, 372)	0.006	566 (400, 712)	494 (383, 800)	0.8
**Glucose/day** (g)	186 (119, 281)	212 (132, 266)	0.7	167 (116, 213)	200 (122, 248)	0.14
**Lipids/day** (g)	19 (11, 35)	23 (10, 33)	0.8	11 (0, 23)	20 (9, 30)	0.087
**Hypocalcemia before TPN**			0.8			0.2
0 -No	84 (77%)	46 (75%)		10 (45%)	16 (64%)	
1 -Yes	25 (23%)	15 (25%)		12 (55%)	9 (36%)	
Unknown	17	18		16	5	
**Hypomagnesemia before TPN**			0.4			0.3
0 -No	95 (87%)	56 (92%)		19 (86%)	18 (72%)	
1 -Yes	14 (13%)	5 (8.2%)		3 (14%)	7 (28%)	
Unknown	17	18		16	5	
**Hypophosphatemia before TPN**			0.2			0.7
0 -No	95 (87%)	49 (80%)		17 (77%)	18 (72%)	
1 -Yes	14 (13%)	12 (20%)		5 (23%)	7 (28%)	
Unknown	17	18		16	5	
**SAA supplementation**			0.3			>0.9
0 -No	58 (46%)	30 (38%)		34 (89%)	27 (90%)	
1 -Yes	68 (54%)	49 (62%)		4 (11%)	3 (10%)	
**EAA supplementation**			0.051			>0.9
0 -No	114 (90%)	64 (81%)		37 (97%)	28 (97%)	
1 -Yes	12 (9.5%)	15 (19%)		1 (2.6%)	1 (3.4%)	
Unknown				0	1	

TPN - total parenteral nutrition; HSCT - hematopoietic stem cell transplantation; SAA – selective amino acids; EAA – essential amino acids.

[1] : Median (IQR); n (%)

[2] : Wilcoxon rank sum test; Pearson's Chi-squared test, Fisher's exact test.

Table S2: Incidence of complication of PN.

	HSCT group			Non - HSCT group		
**Characteristic**	**Males** (n = 126)[1]	**Females,** (n = 79)[1]	**p-value**[2]	**Males** (n = 38)[1]	**Female** (n = 30)[1]	**p-value**[2]
**Hypercalcemia after TPN**			0.4			>0.9
0 -No	106 (100%)	60 (98%)		23 (100%)	23 (100%)	
1 -Yes	0 (0%)	1 (1.6%)		0 (0%)	0 (0%)	
Unknown	20	18		15	7	
**Hypermagnesemia after TPN**			>0.9			0.6
0 -No	107 (100%)	61 (100%)		20 (91%)	22 (96%)	
1 -Yes	0 (0%)	0 (0%)		2 (9.1%)	1 (4.3%)	
Unknown	19	18		16	7	
**Hyperphosphatemia after TPN**			0.028			0.3
0 -No	64 (60%)	47 (77%)		11 (50%)	8 (35%)	
1 - Yes	42 (40%)	14 (23%)		11 (50%)	15 (65%)	
Unknown	20	18		16	7	
**Increased transaminases**			0.3			0.9
0 -No	29 (26%)	21 (33%)		9 (39%)	10 (42%)	
1 -Yes	83 (74%)	43 (67%)		14 (61%)	14 (58%)	
Unknown	14	15		15	6	
**Cholestasis**			0.2			0.5
0 -No	92 (74%)	65 (82%)		36 (95%)	30 (100%)	
1 -Yes	33 (26%)	14 (18%)		2 (5.3%)	0 (0%)	
Unknown	1	0				
**Hyperammonemia**			0.7			0.12
0 -No	116 (92%)	73 (94%)		34 (89%)	30 (100%)	
1 -Yes	10 (7.9%)	5 (6.4%)		4 (11%)	0 (0%)	
Unknown	0	1				
**Hypercholesterolemia**			0.5			0.4
0 -No	101 (80%)	66 (84%)		34 (89%)	29 (97%)	
1 -Yes	25 (20%)	13 (16%)		4 (11%)	1 (3.3%)	
**Hypertriglyceridemia**			0.02			>0.9
0 -No	95 (75%)	70 (89%)		30 (79%)	24 (80%)	
1 -Yes	31 (25%)	9 (11%)		8 (21%)	6 (20%)	
**Hyperglycemia**			0.7			0.3
0 -No	100 (79%)	61 (77%)		32 (84%)	28 (93%)	
1 -Yes	26 (21%)	18 (23%)		6 (16%)	2 (6.7%)	
**Liver damage**			0.6			0.8
0 -No	30 (24%)	16 (20%)		19 (50%)	14 (47%)	
1 -Yes	96 (76%)	63 (80%)		19 (50%)	16 (53%)	
**Metabolic acidosis**			0.3			>0.9
0 -No	120 (95%)	78 (99%)		37 (97%)	30 (100%)	
1 -Yes	6 (4.8%)	1 (1.3%)		1 (2.6%)	0 (0%)	
**Metabolic alkalosis**			0.2			0.12
0 -No	121 (96%)	79 (100%)		34 (89%)	30 (100%)	
1 -Yes	5 (4.0%)	0 (0%)		4 (11%)	0 (0%)	
**Respiratory alkalosis**			>0.9			0.4
0 -No	126 (100%)	79 (100%)		38 (100%)	29 (97%)	
1 -Yes	0 (0%)	0 (0%)		0 (0%)	1 (3.3%)	
**Sepsis**			0.7			0.033
0 -No	109 (87%)	67 (85%)		26 (68%)	27 (90%)	
1 -Yes	17 (13%)	12 (15%)		12 (32%)	3 (10%)	
**CVC thrombosis**			0.2			0.3
0 -No	109 (87%)	63 (80%)		31 (82%)	28 (93%)	

1 -Yes	17 (13%)	16 (20%)	7 (18%)	2 (6.7%)

TPN – total parenteral nutrition; HSCT – hematopoietic stem cell transplantation; CVC – central venous catheter.
[1] Median (IQR); n (%)
[2] Wilcoxon rank sum test; Pearson's Chi-squared test, Fisher's exact test.

Table S3: Linear Multivariate Regression analysis (continuous variables).

	**Total population**			**HSCT group**			**Non-HSCT group**		
**Characteristics**	**Coefficient Estimate**	**st. error**	**p-value**	**Coefficient Estimate**	**st. error**	**p-value**	**Coefficient Estimate**	**st. error**	**p-value**
**Volume/day**									
(Intercept)	1083.55	411.64	0.009	1531.82	467.74	0.001	1111.88	184.55	<0.001
**Gender: F**	366.34	303.65	0.229	444.67	405.35	0.274	175.32	153.25	0.257
TPN duration	-26.41	10.71	0.014	-27.73	12.72	0.030	-12.07	10.57	0.258
Age	95.76	26.83	<0.001	97.02	35.89	0.007	88.77	13.26	<0.001
HSCT: Yes	469.40	353.13	0.185						
**Magnesium/day**									
(Intercept)	56.27	42.58	0.187	133.74	47.64	0.005	90.32	30.67	0.004
**Gender: F**	38.04	31.41	0.227	48.11	41.29	0.245	10.50	25.47	0.682
TPN duration	-1.49	1.11	0.179	-1.72	1.30	0.185	1.23	1.76	0.486
Age	12.86	2.78	<0.001	14.37	3.66	<0.001	8.10	2.20	<0.001
HSCT: Yes	92.84	36.53	<0.001						
**Phophorum/day**									
(Intercept)	262.09	149.88	0.082	277.79	168.96	0.102	268.80	95.00	0.006
**Gender: F**	214.23	110.56	0.054	252.00	146.42	0.087	111.72	78.89	0.162
TPN duration	-4.14	3.90	0.290	-4.22	4.59	0.359	-5.07	5.44	0.355
Age	17.61	9.77	0.073	15.49	12.97	0.234	22.52	6.83	0.002
HSCT: Yes	7.39	128.58	0.954						
**Calcium/day**									
(Intercept)	521.66	167.41	0.002	501.08	189.14	0.009	368.61	89.35	<0.001
**Gender: F**	131.77	123.49	0.287	193.77	163.91	0.239	-2.32	74.20	0.975
TPN duration	-9.28	4.36	0.034	-9.97	5.14	0.054	-3.10	5.12	0.546
HSCT: Yes	-77.09	143.61	0.592	1.06	14.51	0.942	24.72	6.42	0.000
Age	7.87	10.91	0.471						
**Glucose/day**									
(Intercept)	91.42	34.99	0.010	140.75	39.54	<0.001	368.61	89.35	<0.001
**Gender: F**	33.23	25.81	0.199	35.14	34.27	0.306	-2.32	74.20	0.975
TPN duration	-1.79	0.91	0.051	-1.84	1.08	0.088	-3.10	5.12	0.546
Age	10.41	2.28	<0.001	11.06	3.03	0.000	24.72	6.42	<0.001
HSCT: Yes	55.80	30.02	0.064						
**Lipids/day**									
(Intercept)	3.09	7.76	0.691	13.88	8.75	0.114	107.04	20.51	<0.001
**Gender: F**	5.82	5.73	0.310	6.47	7.58	0.395	26.93	17.03	0.119
TPN duration	-0.16	0.20	0.436	-0.23	0.24	0.334	-1.13	1.18	0.338
Age	1.51	0.51	0.003	1.55	0.67	0.022	8.46	1.47	<0.001
HSCT: Yes	10.27	6.66	0.124						

TPN – total parenteral nutrition; HSCT – hematopoietic stem cell transplantation.

Table S4. Multivariate Logistic Regression analysis (categorical variables).

**Characteristic**	1 vs 0	**TOTAL POPULATION OR(95%CI)**	**p-value[1]**	**HSCT-group OR(95%CI)**	**Not HSCT-group OR(95%CI)**
**Hypercalcemia after TPN**					
	**Gender: F vs M**	464091090.63(0,Inf)	0.997	595583903.22(0,Inf)	1(0,Inf)
	Age*	0.59(0.24,1.45)	0.25	0.59(0.24,1.45)	1(0,Inf)
	TPN duration*	1.05(0.83,1.34)	0.674	1.05(0.83,1.34)	1(0,Inf)
	HSCT: 1 vs 0	110558065.03(0,Inf)	0.998		
**Hypermagnesemia after TPN**					
	**Gender: F vs M**	0.49(0.04,6.31)	0.584	1(0,Inf)	0.49(0.04,6.31)
	Age*	0.82(0.59,1.13)	0.222	1(0,Inf)	0.82(0.6,1.13)
	TPN duration*	1.04(0.91,1.18)	0.59	1(0,Inf)	1.04(0.91,1.18)
	HSCT: 1 vs 0	0(0,Inf)	0.995		
**Hyperphosphatemia after TPN**					
	**Gender: F vs M**	0.74(0.4,1.37)	0.331	0.51(0.25,1.06)	2.15(0.57,8.11)
	Age*	0.91(0.86,0.96)	<0.001	0.92(0.87,0.98)	0.86(0.76,0.98)
	TPN duration*	0.98(0.96,1.01)	0.166	0.99(0.96,1.01)	0.94(0.86,1.03)
	HSCT: 1 vs 0	0.45(0.21,0.93)	0.031		
**Increased transaminases**					
	**Gender: F vs M**	0.79(0.44,1.42)	0.432	0.75(0.38,1.49)	0.92(0.28,2.98)
	Age*	0.96(0.91,1.01)	0.133	0.95(0.9,1.01)	0.99(0.89,1.1)
	TPN duration*	1.0036(0.9827,1.0249)	0.74	1.0047(0.9831,1.0269)	0.98(0.88,1.1)
	HSCT: 1 vs 0	1.73(0.84,3.54)	0.137		
**Cholestasis**					
	**Gender: F vs M**	0.51(0.25,1.03)	0.059	0.54(0.26,1.11)	0(0,Inf)
	Age*	1.04(0.98,1.1)	0.215	1.04(0.98,1.11)	0.98(0.76,1.26)
	TPN duration*	1.02(1,1.04)	0.084	1.02(1,1.04)	0.99(0.81,1.2)
	HSCT: 1 vs 0	8.43(1.96,36.21)	0.004		
**Hyperammonemia**					
	**Gender: F vs M**	0.66(0.22,1.99)	0.455	1.15(0.35,3.82)	0(0,Inf)
	Age*	0.89(0.81,0.98)	0.014	0.86(0.77,0.96)	0.29(0.05,1.62)
	TPN duration*	1.05(1.02,1.08)	<0.001	1.04(1.01,1.07)	2.67(0.76,9.39)
	HSCT: 1 vs 0	0.78(0.23,2.69)	0.698		
**SAA supplementation**					
	**Gender: F vs M**	1.14(0.64,2.02)	0.657	1.11(0.6,2.06)	1.43(0.25,8.13)
	Age*	1.09(1.03,1.14)	0.002	1.1(1.04,1.16)	1.01(0.88,1.16)
	PN duration*	1.06(1.03,1.1)	<0.001	1.06(1.02,1.09)	1.1(1.01,1.2)
	HSCT: 1 vs 0	9.02(3.8,21.4)	<0.001		
**EAA supplementation**					
	**Gender: F vs M**	2.16(0.95,4.92)	0.068	2.2(0.93,5.21)	1.96(0.09,43.3)
	Age*	1.01(0.94,1.09)	0.788	1.02(0.94,1.1)	0.96(0.75,1.22)
	TPN duration*	1.03(1.01,1.06)	0.002	1.03(1.01,1.06)	1.1(0.95,1.27)
	HSCT: 1 vs 0	3.6(0.81,16.04)	0.093		
**Hypercholesterolemia**					
	**Gender: F vs M**	0.6(0.29,1.23)	0.165	0.68(0.32,1.45)	0.8(0.05,12.28)
	Age*	1.12(1.05,1.2)	<0.001	1.1(1.02,1.17)	1.45(0.98,2.15)

	TPN duration*	1.02(1,1.04)	0.125	1.01(0.99,1.03)	1.1(0.96,1.27)
	HSCT: 1 vs 0	2.46(0.89,6.76)	0.082		
**Hypertriglyceridemia**					
	**Gender: F vs M**	0.47(0.24,0.91)	0.025	0.34(0.15,0.78)	1.3(0.36,4.72)
	Age*	1.06(1,1.12)	0.05	1.07(1,1.14)	1.03(0.92,1.15)
	TPN duration*	1.02(1,1.04)	0.08	1.01(0.99,1.03)	1.1(1.02,1.19)
	HSCT: 1 vs 0	0.75(0.37,1.55)	0.442		
**Hyperglycemia**					
	**Gender: F vs M**	0.81(0.42,1.57)	0.534	0.95(0.47,1.94)	0.52(0.08,3.55)
	Age*	1.13(1.06,1.21)	<0.001	1.1(1.03,1.18)	1.33(1.05,1.68)
	TPN duration*	1.03(1.01,1.05)	0.013	1.02(1,1.05)	1.04(0.93,1.16)
	HSCT: 1 vs 0	1.6(0.68,3.75)	0.285		
**Liver damage**					
	**Gender: F vs M**	1.13(0.64,2)	0.666	1.07(0.53,2.17)	1.44(0.52,3.98)
	Age*	1.04(0.99,1.09)	0.165	1.06(0.99,1.13)	0.98(0.9,1.08)
	TPN duration*	1.04(1.01,1.07)	0.019	1.03(0.99,1.06)	1.12(1.02,1.23)
	HSCT: 1 vs 0	2.57(1.39,4.72)	0.002		
**Metabolic acidosis**					
	**Gender: F vs M**	0.28(0.03,2.36)	0.241	0.37(0.04,3.33)	0(0,Inf)
	Age*	0.84(0.71,0.99)	0.037	0.75(0.59,0.95)	1.24(0.78,1.97)
	TPN duration*	1.0059(0.9651,1.0485)	0.779	1.0063(0.9646,1.0497)	0.84(0.47,1.49)
	HSCT: 1 vs 0	2.37(0.27,20.97)	0.438		
**Metabolic alkalosis**					
	**Gender: F vs M**	0(0,Inf)	0.994	0(0,Inf)	0(0,Inf)
	Age*	0.81(0.69,0.97)	0.02	0.79(0.61,1.03)	0.28(0.06,1.28)
	TPN duration*	1.05(1.02,1.09)	0.002	1.04(1.01,1.08)	2.32(0.87,6.16)
	HSCT: 1 vs 0	0.15(0.03,0.82)	0.029		
**Respiratory alkalosis**					
	**Gender: F vs M**	92929997.48(0,Inf)	0.998	1(0,Inf)	72643917.92(0,Inf)
	Age*	1.46(0.59,3.63)	0.411	1(0,Inf)	1.46(0.59,3.63)
	TPN duration*	0.64(0.16,2.51)	0.525	1(0,Inf)	0.64(0.16,2.51)
	HSCT: 1 vs 0	0(0,Inf)	0.998		
**Sepsis**					
	**Gender: F vs M**	0.72(0.36,1.46)	0.366	1.33(0.57,3.14)	0.26(0.06,1.09)
	Age*	0.99(0.93,1.05)	0.722	0.92(0.86,1)	1.11(0.99,1.24)
	TPN duration*	1.04(1.02,1.06)	<0.001	1.04(1.02,1.06)	1.04(0.96,1.13)
	HSCT: 1 vs 0	0.39(0.18,0.82)	0.014		
**CVC thrombosis**					
	**Gender: F vs M**	1.15(0.59,2.25)	0.686	1.69(0.79,3.63)	0.3(0.05,1.66)
	Age*	1.01(0.95,1.08)	0.648	0.98(0.92,1.05)	1.12(0.97,1.29)
	PN duration*	0.9971(0.9727,1.022)	0.815	0.9948(0.9679,1.0224)	0.99(0.8955,1.0946)
	HSCT: 1 vs 0	1.29(0.57,2.92)	0.548		
**Hyperammonemia**					
	**Gender: F vs M**	0.63(0.2,1.93)	0.404	1.08(0.32,3.65)	0(0,Inf)
	Age*	0.88(0.8,0.97)	0.008	0.86(0.77,0.96)	0.27(0.05,1.45)
	TPN duration*	1.05(1.02,1.08)	<0.001	1.04(1.01,1.07)	2.99(0.8,11.15)
	**AAE suppl. : 1 vs 0**	1.73(0.43,7)	0.456	2.05(0.5,8.36)	0(0,Inf)
	HSCT: 1 vs 0	0.74(0.21,2.56)	0.641		

**Cholestasis**					
	**Gender: F vs M**	0.45(0.22,0.95)	0.035	0.48(0.22,1.01)	0(0,Inf)
	Age*	1.06(0.99,1.13)	0.085	1.07(1,1.14)	0.9(0.55,1.5)
	TPN duration*	1.02(1,1.04)	0.078	1.02(1,1.04)	0.95(0.73,1.24)
	HSCT: 1 vs 0	9.09(2.08,39.8)	0.003		
	**Lipids/die (stand.)***	2.66(1.26,5.59)	0.01	2.76(1.26,6.01)	4.85(0.05,485.39)
	**Glucose/day (stand.)***	0.32(0.12,0.89)	0.028	0.31(0.11,0.88)	1.55(0,3669.75)
**Hypertriglyceridemia**					
	**Gender: F vs M**	0.45(0.23,0.88)	0.019	0.31(0.13,0.73)	1.27(0.32,4.96)
	Age*	1.05(0.99,1.12)	0.079	1.07(1,1.15)	1.0087(0.8794,1.1571)
	TPN duration*	1.02(1,1.04)	0.075	1.01(0.99,1.03)	1.13(1.03,1.24)
	HSCT: 1 vs 0	0.72(0.35,1.49)	0.376		
	**Lipids/day (stand.)***	1.38(0.84,2.26)	0.198	1.63(0.87,3.07)	0.41(0.06,2.68)
	**Glucose/day (stand.)***	0.86(0.46,1.6)	0.635	0.69(0.29,1.6)	3.35(0.52,21.67)
**Hypercholesterolemia**					
	**Gender: F vs M**	0.54(0.26,1.14)	0.105	0.61(0.28,1.34)	1.03(0.05,21.95)
	Age*	1.11(1.03,1.19)	0.003	1.08(1.01,1.16)	1.41(0.91,2.19)
	TPN duration*	1.02(1,1.04)	0.077	1.02(0.99,1.04)	1.16(0.96,1.4)
	HSCT: 1 vs 0	2.17(0.78,6.03)	0.138		
	**Lipids/day (stand.)***	1.19(0.75,1.89)	0.462	1.17(0.73,1.87)	0.94(0.08,10.75)
	**Glucose/day (stand.)***	1.24(0.84,1.85)	0.283	1.24(0.83,1.85)	16.68(0.22,1288.5)

OR – odds ratio; TPN - total parenteral nutrition; HSCT - hematopoietic stem cell transplantation; SAA – selective amino acids; EAA – essential amino acids; CVC – central venous catheter.

*: continuous variable.

[1]: p-value Wald's test, only shown for the total population.
